# Eco Assist Techniques through Real-time Monitoring of BEV Energy Usage Efficiency

**DOI:** 10.3390/s150714946

**Published:** 2015-06-25

**Authors:** Younsun Kim, Ingeol Lee, Sungho Kang

**Affiliations:** Department of Electrical and Electronic Engineering, Yonsei University, Seoul 120-749, Korea; E-Mails: younsunny.kim@gmail.com (Y.K.); keor@soc.yonsei.ac.kr (I.L.)

**Keywords:** fuel economy, eco driving, eco guide, energy usage efficiency

## Abstract

Energy efficiency enhancement has become an increasingly important issue for battery electric vehicles. Even if it can be improved in many ways, the driver’s driving pattern strongly influences the battery energy consumption of a vehicle. In this paper, eco assist techniques to simply implement an energy-efficient driving assistant system are introduced, including eco guide, eco control and eco monitoring methods. The eco guide is provided to control the vehicle speed and accelerator pedal stroke, and eco control is suggested to limit the output power of the battery. For eco monitoring, the eco indicator and eco report are suggested to teach eco-friendly driving habits. The vehicle test, which is done in four ways, consists of federal test procedure (FTP)-75, new european driving cycle (NEDC), city and highway cycles, and visual feedback with audible warnings is provided to attract the driver’s voluntary participation. The vehicle test result shows that the energy usage efficiency can be increased up to 19.41%.

## 1. Introduction

As global emission levels and populations continue to rise, the demand for fuel-efficient vehicles has increased greatly. Eco driving can be a good solution to enable energy conservation, reduce carbon dioxide emissions, and contribute to the effort to reduce global warming. Because of the ever-increasing awareness, eco driving techniques have slowly been making their way into the automotive industry for many years and have recently picked up significant momentum as globalization has expanded industry and public horizons [1,2,3,4,5]. Eco driving techniques help a driver or vehicle to operate in a more efficient manner and to reduce fuel consumption; however, they ultimately rely on the driver to take action to improve the energy efficiency [6,7,8].

Eco driving techniques designed to improve fuel economy vary from on-board trip computers to powertrain control solutions, and utilize the sensory information from sensors embedded within vehicle systems to monitor the in-vehicle or out-of-vehicle status [9,10]. These can be segmented into active or passive methods according to the level of vehicle controllability. Active methods enable autonomous control over vehicle systems, while passive methods provide information and guidance based-on the analysis of sensory information obtained from the vehicle. Passive solutions interpret the gathered data and provide some indication for the driver’s action based on their interpretation of sensory data, and active solutions can quickly and efficiently optimize the vehicle system by limiting or eliminating human input [11,12,13].

Eco driving techniques can be categorized into three groups: Eco monitoring, eco guide and eco control. Eco monitoring is used to reflect the instant, historical, and time-elapsed fuel economy, and is used in the auto industry through on-board trip computers [12,13]. The eco guide gives eco assist information using the eco indicator or to provide the eco routing guide using navigation maps to minimize traffic signals, hills and stops [7,8,9]. Eco control minimizes the human influence over the power train while driving. One popular application today is the automatic start-stop system. It deactivates the engine when the vehicle is stopped at a traffic signal and automatically restarts without any driver input when the driver begins to accelerate [5,6].

Recently, the increasing emphasis on fuel economy and emissions has accelerated the need for more sophisticated applications, increased the functionality of on-board trip computers, and required deeper analysis [14,15]. Since fuel economy is maximized when acceleration and braking are minimized, energy usage efficiency can be increased by simply applying eco driving techniques to anticipate what is happening ahead, to drive in such a way so as to minimize acceleration and braking, to ensure cruising at the optimal speed, and to maximize the coasting time at stops [5,6]. In fact, fuel economy not only depends on various physical factors such as road-traffic vehicle conditions and, driving styles, but also influences the vehicle emissions and energy consumption [7,8]. According to previous research, the potential for improving the energy usage efficiency by monitoring the driver’s driving style and analyzing the driver’s behavior and preferences is estimated to be in the range of 20% to 40% [16]. Moreover, fuel economy can be improved relatively easily and inexpensively by changing the driving style [17,18,19,20].

This paper proposes a novel approach to analyze the in-vehicle sensory information from the battery electric vehicle and explores eco assist techniques to enhance energy usage efficiency and to enlarge the driving range. We have analyzed the correlation between the vehicle speed and battery power consumption, investigated the fuel saving behavioral aspects of the accelerator pedal stroke (APS), vehicle speed and battery power consumption, and proposed eco assist techniques to simply implement an energy-efficient driving assistant system. Vehicle control conditions required for economic energy consumption are analyzed, and an eco guide to attract the driver’s voluntary participation is provided using visual feedback with audible warnings.

## 2. Eco Assist Techniques

### 2.1. Analysis of BEV’s Energy Usage Efficiency

For a gasoline vehicle, the fuel consumption is the amount of fuel used per unit distance, such as liters per kilometer (L/km). The fuel economy or efficiency is the distance travelled per unit volume of fuel used, and is measured in kilometers per liter (km/L) or miles per gallon (MPG). For an electric vehicle, the energy consumption is the amount of battery energy used per unit distance (Wh/km), and the energy efficiency is the distance travelled per unit volume of battery energy used (km/Wh). Generally, energy efficiency is one of the vehicle’s unique characteristics, and it depends heavily on the characteristic engine map [21]. In real-world driving situations, the energy consumption and efficiency depend on various physical factors related to the road-traffic-vehicle conditions. In addition, driving styles strongly influence energy consumption for the same physical driving conditions. In this paper, energy efficiency is improved by changing the driver behavior, so in order to reflect the energy efficiency enhancement according to driving pattern, the energy usage efficiency considers both road-traffic-vehicle conditions and driving styles.

**Figure 1:**
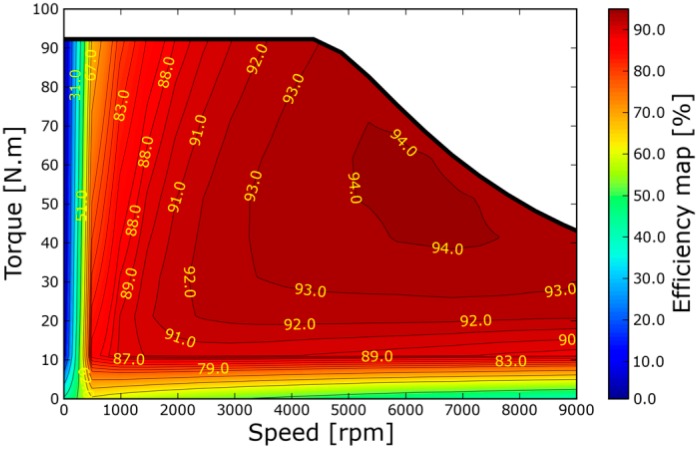
An example of electric engine characteristic map.

The energy efficiency of a battery electric vehicle (BEV) can be defined by analyzing the correlation for some energy saving behavioral aspects: The acceleration, vehicle speed and battery power consumption. For combustion engines, comparatively high acceleration even saves fuel, since the engine operations are generally more efficient for higher power demands. Regarding electric engines, the energy efficiency, which is the conversion factor from the chemical energy to the delivered mechanical energy, depends on the engine speed and power demand, so pure physics rules: Given a fixed maximum speed, the required mechanical energy to attain a certain speed is essentially independent of the acceleration. The efficiency characteristic map of electrical motors is of the order of 90% in most operating conditions, and is essentially flat as shown in Figure 1 [22]. A small differential due to wind drag can be neglected in city traffic.

**Figure 2:**
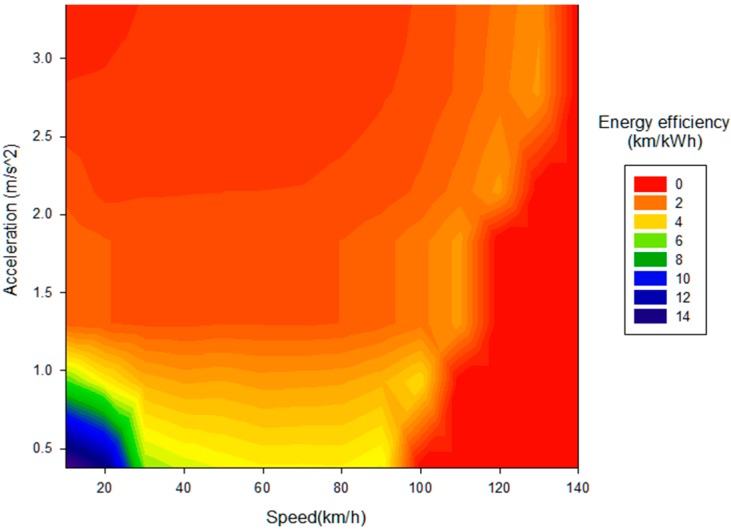
Energy efficiency according to vehicle speed and acceleration.

**Figure 3:**
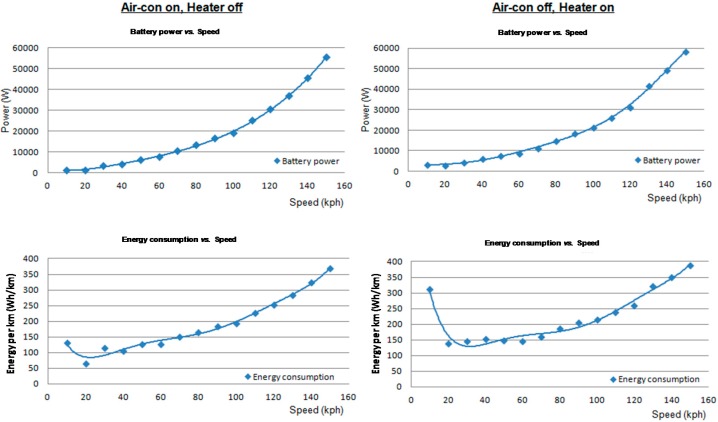
Energy consumption according to vehicle speed.

For an eco assist technique to be of use on the road, real-time monitoring considering various physical factors is very important, so the sensory information of an electric vehicle is collected and the energy efficiency is investigated by profiling an electric motor’s characteristics in real-time. According to our research, the vehicle speed has a large impact on the energy usage efficiency and the acceleration acts as a relevant exogeneous variable as shown in Figure 2. There exists a specific period of vehicle speed, that has high-energy usage efficiency and shows different characteristics under battery energy usage conditions. We conclude that 20 km/h–60 km/h is the most economic speed range when the air-conditioner is off and the heater is on, as shown in Figure 3. On the other hand, the energy usage efficiency tends to increase with the amount of brake pedal strokes, since the state of charge (SOC) of the battery increases because of regenerative braking properties. Therefore, there is no benefit to providing eco guide information for the braking behavior of a battery electric vehicle, whereas there is a benefit for a gasoline-powered vehicle.

### 2.2. Eco Guide

In this paper, the eco guide, including the eco start, eco acceleration and eco speed, is proposed as an eco assist technique for the driver to be able to control the vehicle speed and accelerator pedal stroke. To provide an efficient eco driving guide to the driver, it is necessary to establish distinct criteria based on vehicle-specific and situation-specific data obtained from a large number of sensors, such as the accelerator pedal sensor, vehicle speed sensor and throttle sensor. This sensory information should be monitored and analyzed in real-time, and advice should be generated through the decision making, to let the driver know if the current driving pattern is suitable for energy efficient driving. All eco guides are given when a driver breaks an eco guide rule for more than 3 s, and are monitored to make sure the driver keeps the eco guide rule for over 5 s. Most of all, all eco guides should be provided using visual feedback with audible warnings, and should be personalized to attract the driver’s voluntary participation. Moreover, the visual interface should be intuitive so that all of the eco guides are easily understood, and eco feedback should be instantly given to the driver through a real-time embedded system.

According to our research, the energy efficiency according to the accelerator pedal stroke and vehicle speed period, as shown in Table 1, is valuable information for defining the criteria for the eco start and eco acceleration guide. We have classified the energy efficiency into six levels, and have created eco guides to get a higher energy efficiency level for each speed period. In the case of an eco start guide, it is crucial for the level of the accelerator pedal stroke to stay under 30% when the vehicle speed reaches up to 10 km/h. For an eco acceleration guide, it is recommended that the vehicle acceleration rate should be maintained below 5 m/s^2^ as shown in Table 2. On a road with up-down slopes, it is necessary to anticipate the future states of the vehicle and the information regarding the road gradient in advance, and a study to determine the optimal energy efficiency is under investigation. For the eco speed guide, the energy consumption according to the vehicle speed, as shown in Figure 3, helps to establish the criteria. It is essential to specify the maximum and minimum speed limits according to each driving mode (city or highway) for an eco speed guide. We have defined the most economic speed range for each battery energy condition, and have made eco guides for the driver to follow given the energy saving rules.

**Table 1:** Energy efficiency according to vehicle speed and accelerator pedal stroke (APS).

Energy Efficiency (km/kWh)								
**Speed (km/h)**	**APS 10%**	**APS 15%**	**APS 20%**	**APS 25%**	**APS 30%**	**APS 35%**	**APS 40%**	**APS 45%**
0~10	15.91696	6.557377	1.804124	1.804124	1.511335	0.819672	0.570342	0.46225
10~20	13.69863	4.847397	1.621622	1.621622	1.079137	0.846561	0.614125	0.55814
20~30	6.021898	3.474903	1.486014	1.486014	1.152263	0.91135	0.784314	0.613497
30~40	5.480924	3.150599	1.474359	1.474359	1.173564	0.889152	0.776119	0.651466
40~50	5.158307	3.230148	1.589528	1.587302	1.19225	0.949668	0.777832	0.675676
50~60	4.871673	3.031324	1.553398	1.539085	1.20075	0.988924	0.838095	0.680787
60~70	4.891942	3.081828	1.530612	1.542734	1.212938	0.967742	0.818063	0.721311
70~80	4.850118	3.144394	1.605442	1.605442	1.294149	1.019541	0.844457	0.712401
80~90	5.283276	3.514281	1.845419	1.854749	1.387256	1.158491	0.987269	0.782677
90~100	-	4.344299	2.257298	2.234637	1.679861	1.306512	1.217565	0.949768
100~110	-	-	2.938327	2.936795	2.186712	1.669097	1.621356	1.196215
110~120	-	-	-	-	2.929261	2.241953	2.069199	1.623244
120~130	-	-	-	-	-	2.904916	2.668639	2.028933
130~140	-	-	-	-	-	-	-	2.489998

**Table 2:** Acceleration according to vehicle speed and APS.

Acceleration (m/s^2^)								
**Speed(km/h)**	**APS 10%**	**APS 15%**	**APS 20%**	**APS 25%**	**APS 30%**	**APS 35%**	**APS 40%**	**APS 45%**
0~10	0.375115	0.926312	1.304114	1.833473	2.149234	2.791978	3.555366	3.362509
10~20	0.652061	1.439458	2.275701	3.232971	4.077705	4.97008	6.128653	6.749961
20~30	1.042836	1.974302	3.02712	4.179851	5.257845	6.403935	7.510778	8.612078
30~40	0.955976	1.902826	2.946984	4.077489	5.230235	6.362902	7.511737	8.6123
40~50	0.861198	1.790279	2.814111	3.917636	5.020055	6.243756	7.345757	8.394896
50~60	0.747745	1.676193	2.707254	3.812676	4.897136	6.054442	7.239085	8.325008
60~70	0.622423	1.541573	2.554983	3.646215	4.757102	5.911388	6.985972	8.185651
70~80	0.480282	1.383696	2.389777	3.480537	4.624256	5.774506	6.91343	8.124335
80~90	0.236782	1.013174	1.89213	2.887203	3.980511	5.201993	5.893655	7.291819
90~100	-	0.451013	1.165681	1.982255	2.964412	4.09487	4.440004	5.946576
100~110	-	-	0.399277	1.075342	1.863763	2.838071	2.973226	4.306076
110~120	-	-	-	-	0.85751	1.603415	1.734356	2.759626
120~130	-	-	-	-	-	0.636716	0.773218	1.539295
130~140	-	-	-	-	-	-	-	0.714077

### 2.3. Eco Control

In this paper, eco control is used to limit the output power of the battery as an eco assist technique. The output power of the battery is limited to 12 kW in hard mode and 18 kW in soft mode. In soft mode, the power limitation is dynamically allocated according to the vehicle speed. It is permitted up to 12 kW at low speed and 18 kW at high speed. This is provided by modifying the electronic control unit (ECU) of the vehicle control module (VCM). A study of enriched map data, such as the manipulation of the throttle when traveling up or downhill, or facilitating or preventing a gear change when entering a particularly curvy area of the road, is under investigation.

### 2.4. Eco Monitoring

**Figure 4:**
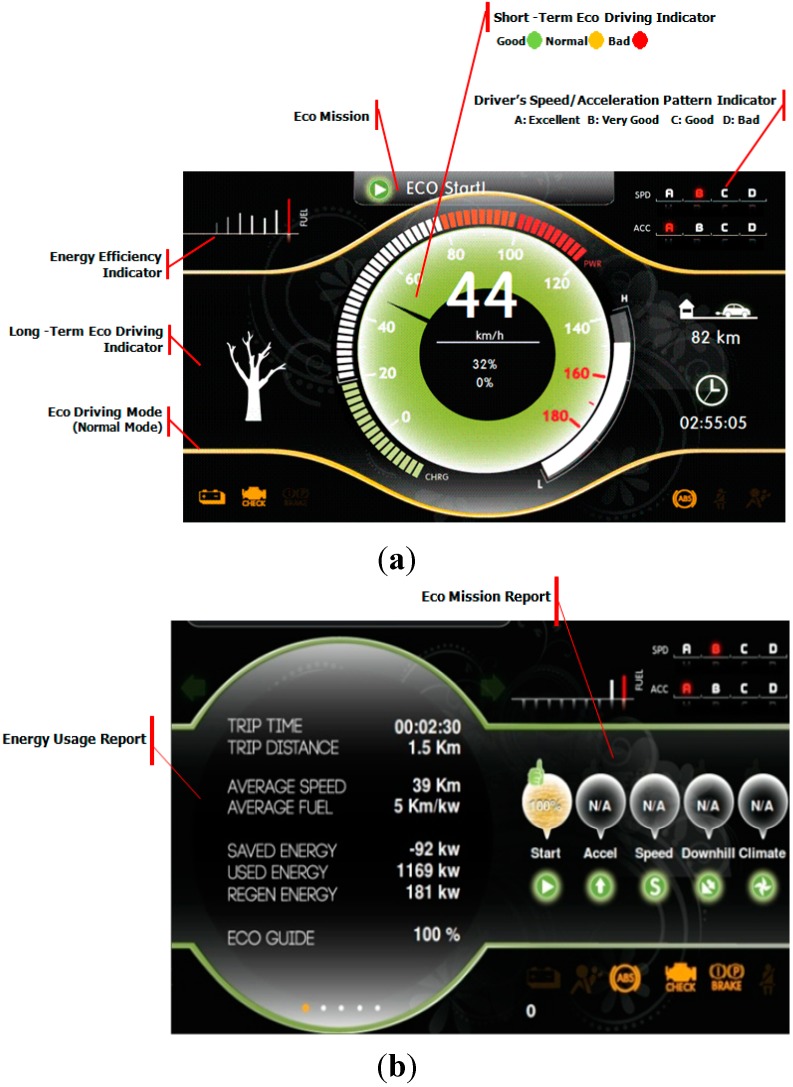
(**a**) Eco indicator; (**b**) Eco report.

As eco monitoring methods, the eco indicator and eco report are proposed as eco assist techniques. A short-term indicator is created to change the color (red, yellow and green) according to the level of energy usage efficiency. It gives the driver the current status information when excessive acceleration is detected or the vehicle speed exceeds the limit of a specific speed range in terms of the electric motor’s energy efficiency. A long-term indicator is provided by displaying a tree’s growth process to help drivers acquire intuitive eco-friendly driving habits. An energy usage efficiency indicator is used to provide the history of the driver’s energy usage patterns with the driver’s speed or acceleration pattern indicator as shown in Figure 4.

In this paper, the eco guide is suggested when the driver’s acceleration pattern or speed pattern is critical for energy usage efficiency. The frequency of the eco guide is controlled according to the driver’s preferences. In the case of the eco guide, it is significant to understand the driver’s tendencies, and therefore the number of eco guides is increased or decreased according to the propensity for eco driving. An eco mission report, which shows how much the driver follows the provided eco guides while driving, is provided in order for the driver to be able to do self-checking while driving or after driving. The energy usage report is offered at the end of driving with the eco mission report. It allows the driver to learn self-driving habits and to improve the energy usage efficiency through personalized advice.

## 3. Experimental Results

### 3.1. Real-Time Embedded System

**Figure 5:**
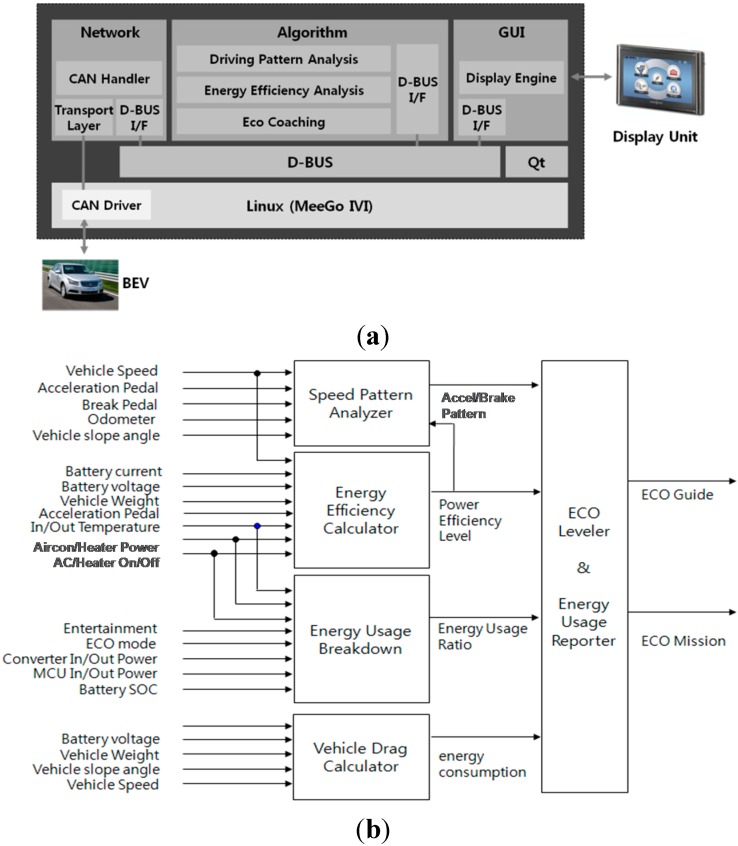
(**a**) Real-time embedded system; (**b**) Block diagram for algorithms.

A real-time embedded system is developed using the Freescale i.MX6 ARD reference board as shown in Figure 5a. Ubuntu-based Linux is used as the operating system, and the GUI implementation is done with the Qt programming tool. All of the algorithms, which are comprised of driving pattern analysis, energy efficiency analysis and eco coaching, are implemented using the C and C++ programming languages. Since this sensory information is limitedly obtained from an on-board diagnostics port such as OBDII, almost all sensory information is acquired from the VCM. The CAN Bus channel is used to get information for the vehicle dynamics state, and it is not only capable of reading the various vehicular parameters, but also of detecting variables from the vehicle environment by using several vehicle sensors in real-time. The monitored features cover the battery current, battery voltage, vehicle speed, accelerator pedal stroke, brake pedal stroke and other vehicle dynamics information as shown in Figure 5b.

### 3.2. Test Method

**Figure 6:**
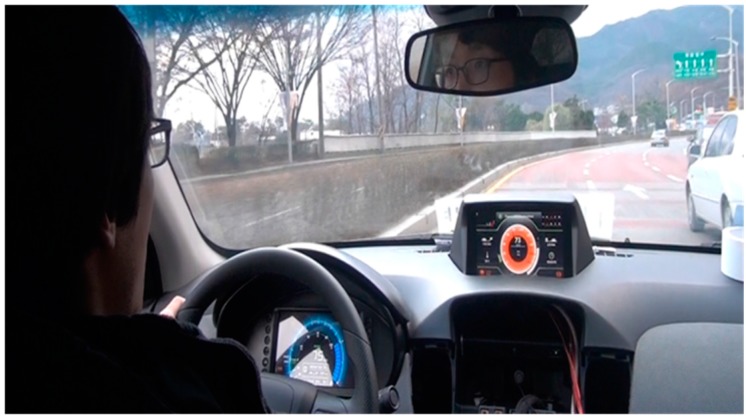
Vehicle test for energy usage efficiency enhancement.

The energy usage efficiency enhancement according to the proposed eco assist techniques is evaluated through real vehicle testing using the Vista 1.0 electric vehicle model as shown in Figure 6. The vehicle test is done in four different ways: Using the FTP-75 and NEDC driving cycles at the Dynamo Lab, and during a city driving cycle and a highway driving cycle on real roads. We have made our own profiles for the city driving cycle on a virtually real road, and for the highway driving cycle on a real road, but they were not sufficient. In a real-world experiment, there is always the danger of an accident during the test, and therefore it is impossible to control these factors for our convenience while driving. The driving time is too short in the city driving cycle, and it is not easy to emulate speed, acceleration, and deceleration patterns under the same driving conditions in the highway driving cycle. To establish our own driving cycles, we used the NEDC and FTP-75 driving cycles, which are officially used for emission certification and fuel economy testing of light-duty vehicles in the USA and Europe. These regulatory driving cycle tests are conducted using a dynamometer, which simulates “typical” trips in the city or on the highway.

**Figure 7:**
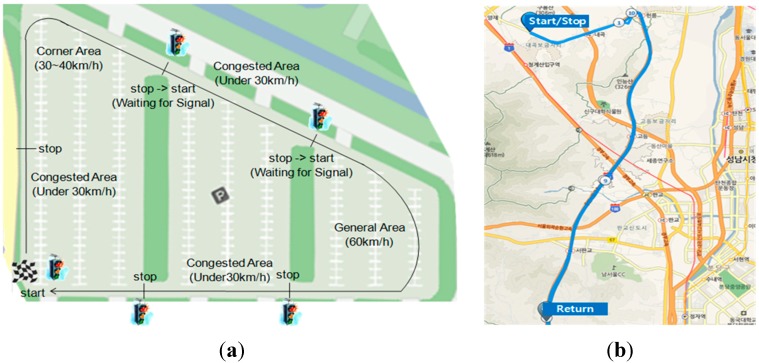
(**a**) City driving cycle; (**b**) Highway driving cycle.

In the case of the city driving cycle, the vehicle level test is done in a large empty parking lot using a pre-defined driving profile, which has a general area, a corner area, and a congested area as shown in Figure 7a. A road of about 2.5 km in length and 5.5 min in driving time, which was located in Seoul, South Korea, is used as the testing route. Each area limits the maximum vehicle speed, a vehicle start-stop condition exists between one area and the other area, and a repetitive test per one round turn is performed with a pre-defined driving cycle. For the highway driving cycle, the highway test, which has a total driving distance of 34.7 km and takes 32 min of driving time, is executed as shown in Figure 7b.

### 3.3. Test Result

**Figure 8:**
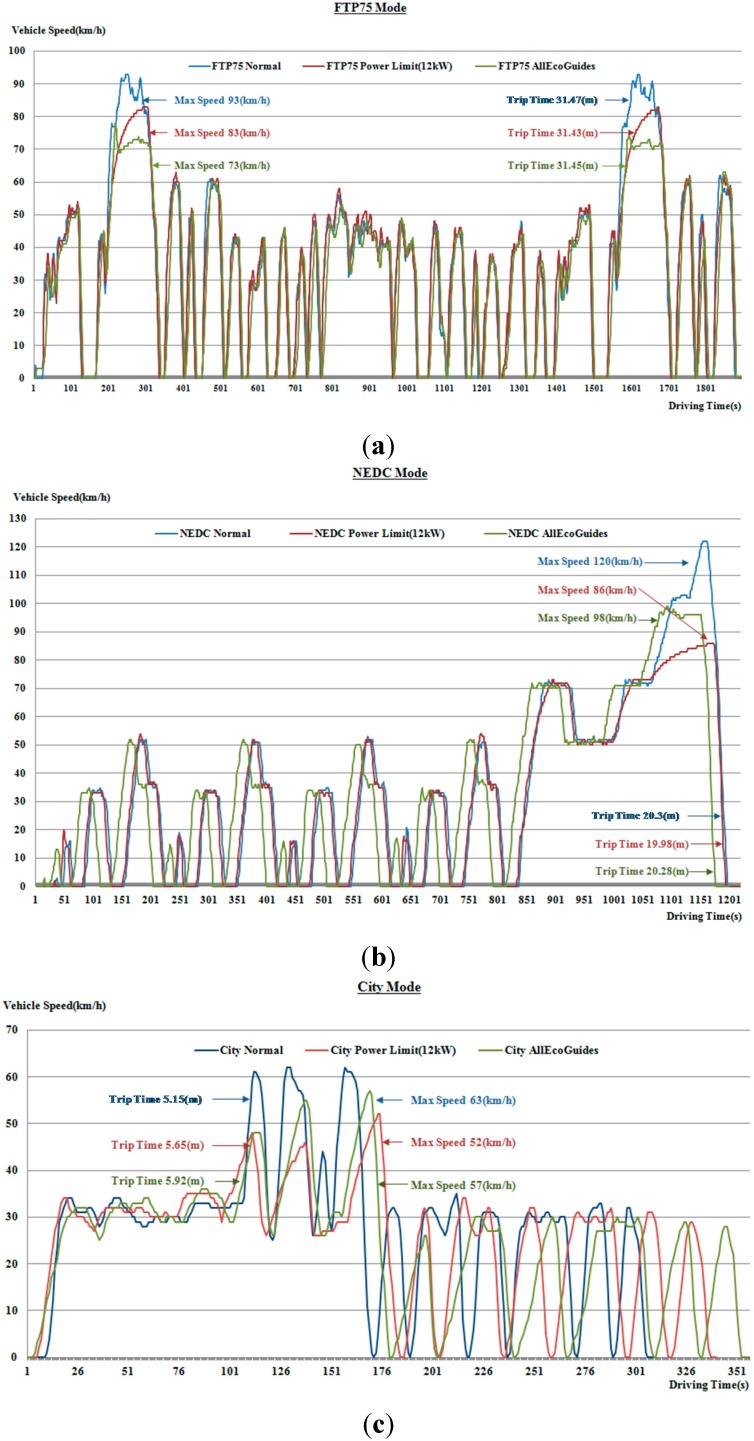

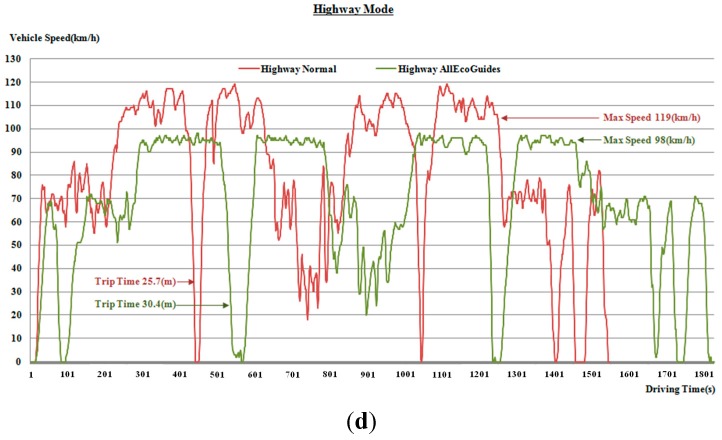
Test result (**a**) FTP-75 mode; (**b**) NEDC mode; (**c**) City mode; (**d**) Highway mode.

In this paper, eco guide, eco control and eco monitoring methods are used as eco assist techniques, and the energy usage efficiency is improved by changing the driver behavior. The eco guide is provided for the driver to be able to control the vehicle speed and accelerator pedal stroke, and eco control is suggested to limit the output power of the battery without any driver input. For eco monitoring, the eco indicator and eco report are used to learn eco-friendly driving habits. The vehicle test result shows that the energy usage efficiency can be increased over 7% for the FTP-75 and NEDC driving cycles, and over 17% in the city and highway driving cycles when the driver adheres to all eco guides as shown in Figure 8 and Table 3. With respect to the regulatory driving cycles, they are standardized and prescribe certain speed and acceleration profiles, so we could not fulfill the specifications regarding maximum speed, average speed and trip distance.

**Table 3:** Test result of energy usage efficiency enhancement.

Eco Assist Techniques			Trip Distance (m)	Average Speed (km/h)	Used Energy (W)	Regen. Energy (W)	Average Energy Efficiency (km/kWh)	Energy Usage Efficiency Increase (%)
FTP-75	FTP-75 Normal		18347	35.24	11782	1612	5.61	100.00
	Eco Control	Hard Mode(12 kW)	18100	34.77	9817	1198	6.63	118.41
		Soft Mode(18 kW)	19023	36.54	10576	1361	6.37	113.67
	Eco Guide	All Guide	17259	33.15	10336	1552	6.00	107.14
NEDC	NEDC Normal		11309	44.93	7153	598	5.69	100.00
	Eco Control	Hard Mode(12 kW)	10689	42.15	5839	579	6.53	114.85
		Soft Mode(18 kW)	10985	43.33	6396	611	6.18	108.61
	Eco Guide	All Guide	10936	42.16	6303	531	6.24	109.74
City	City Normal		2432	29.75	2377	257	3.68	100.00
	Eco Control	Hard Mode(12 kW)	2441	27.79	2081	176	4.22	114.67
		Soft Mode(18 kW)	2440	28.92	2202	222	3.98	108.15
	Eco Guide	All Guide	2419	26.01	2020	206	4.31	117.12
Highway	Highway Normal		34737	83.79	30223	1600	4.13	100.00
	Eco Guide	All Guide	34718	71.21	25296	1422	4.94	119.41

It is found that when the test is done in highway mode, for a trip distance of about 34.73 km, 30.22 kW of battery energy is consumed, which yields an average energy usage efficiency of 4.13 km/kWh. When all eco guides are applied, only 25.29 kW of battery energy is consumed for a trip distance of 34.71 km, which yields an average energy usage efficiency of 4.94 km/kWh. A 19.41% improvement in the average energy usage efficiency is achieved. In this case, even if the comparison test is done with the same trip distance, there are differences in the maximum speeds (119 km/h for normal, 98 km/h for eco driving) and the total trip times (25.7 min for normal, 30.4 min for eco driving). In the case of an all-electric vehicle, energy recuperation occurs during deceleration, and the kinetic energy of the vehicle will continue to turn the electric engine, so what matters is the maximum speed, which is probably also responsible for the observed increase in the energy usage efficiency. This is mainly due to round-turn losses during the recuperation phase. When it comes to applying eco control, the energy usage efficiency improvement depends on the output power limitation, and therefore the hard mode shows better enhancement than the soft mode by an average of 5.83%. The output power of the battery is restricted by force, so the eco control test cannot be performed for the highway driving cycle.

## 4. Conclusions

In this paper, eco assist techniques for an energy-efficient driving assistant system are provided by looking into the fuel saving behavioral aspects of the acceleration, vehicle speed and battery power consumption. A real-time embedded system is developed to obtain information regarding the vehicle dynamics state, to analyze the energy usage efficiency enhancement, and to provide eco assist techniques. The understanding of the eco guide based on the driver’s characteristics is considered to create positive user experiences, and an eco mission with visual feedback and audible warnings is designed to adapt to the driver’s eco driving styles. An energy usage efficiency enhancement test is done using a Vista 1.0 electric vehicle model in four different ways: An FTP-75 cycle, NEDC cycle, city cycle, and highway cycle. The test results show that the energy usage efficiency can be increased up to 19.41% by using the proposed eco assist techniques. Specifically, the energy usage efficiency can be increased over 7% in the FTP75 and NEDC driving cycles, and over 17% in city and highway driving cycles when the driver adheres to all eco guides.
